# Supplementary figures and images for: Proinflammatory cytokines and ARDS pulmonary edema fluid induce CD40 on human mesenchymal stromal cells—A potential mechanism for immune modulation
Source: PLoS One. 2020 Oct 6;15(10):e0240319. doi: 10.1371/journal.pone.0240319 (PMC7537876; doi:10.1371/journal.pone.0240319)

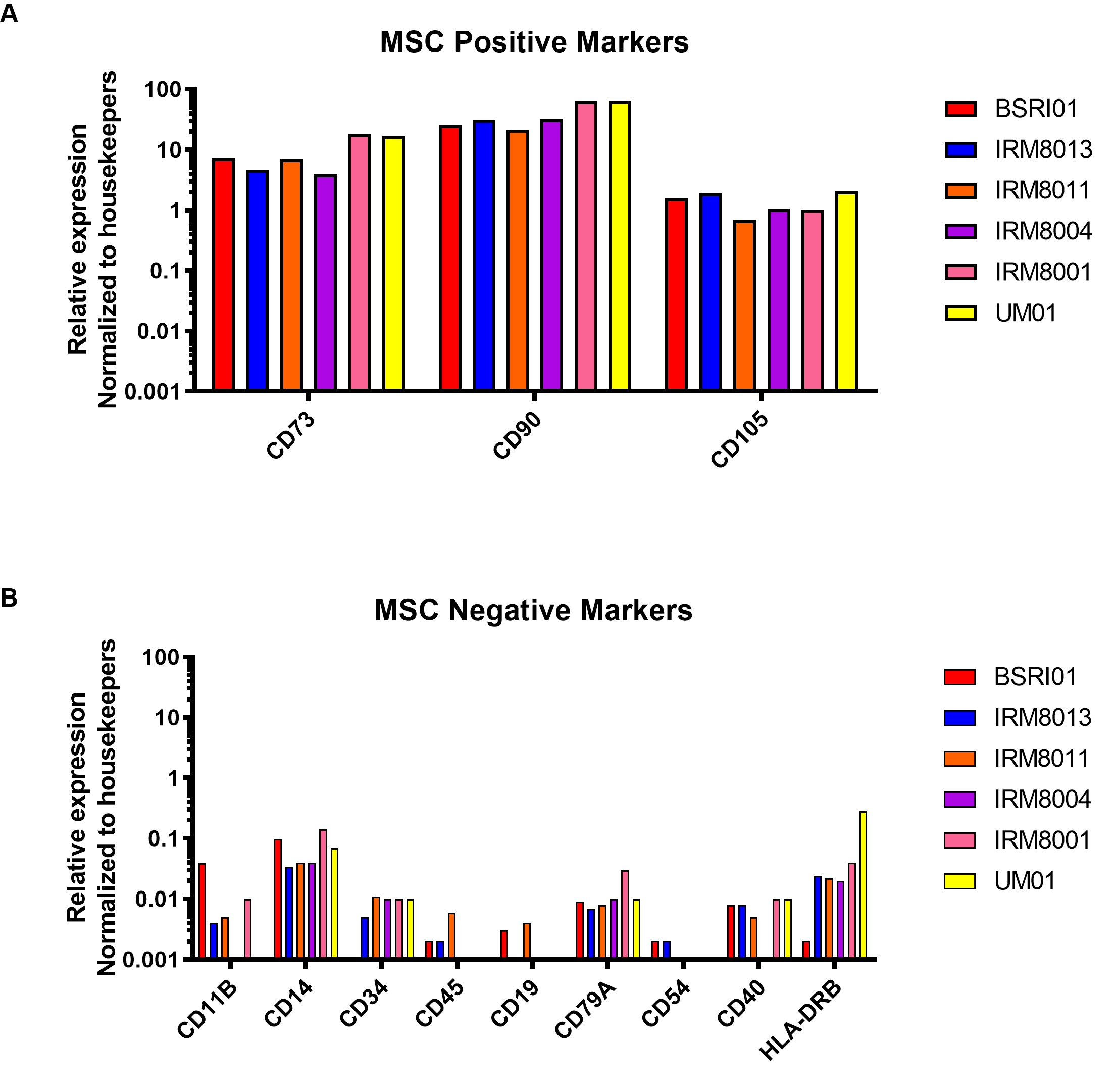

Supplement: S1 Fig — (A) All hMSC cell lines expressed CD73, CD90, and CD105 and (B) did not express high levels of CD11, CD14, CD34, CD45, CD19, CD79A, CD54, CD40, or HLA-DRB. mRNA expression levels were normalized to housekeeper genes EIF2E2 and TBP. (TIF) [file pone.0240319.s001.tif]

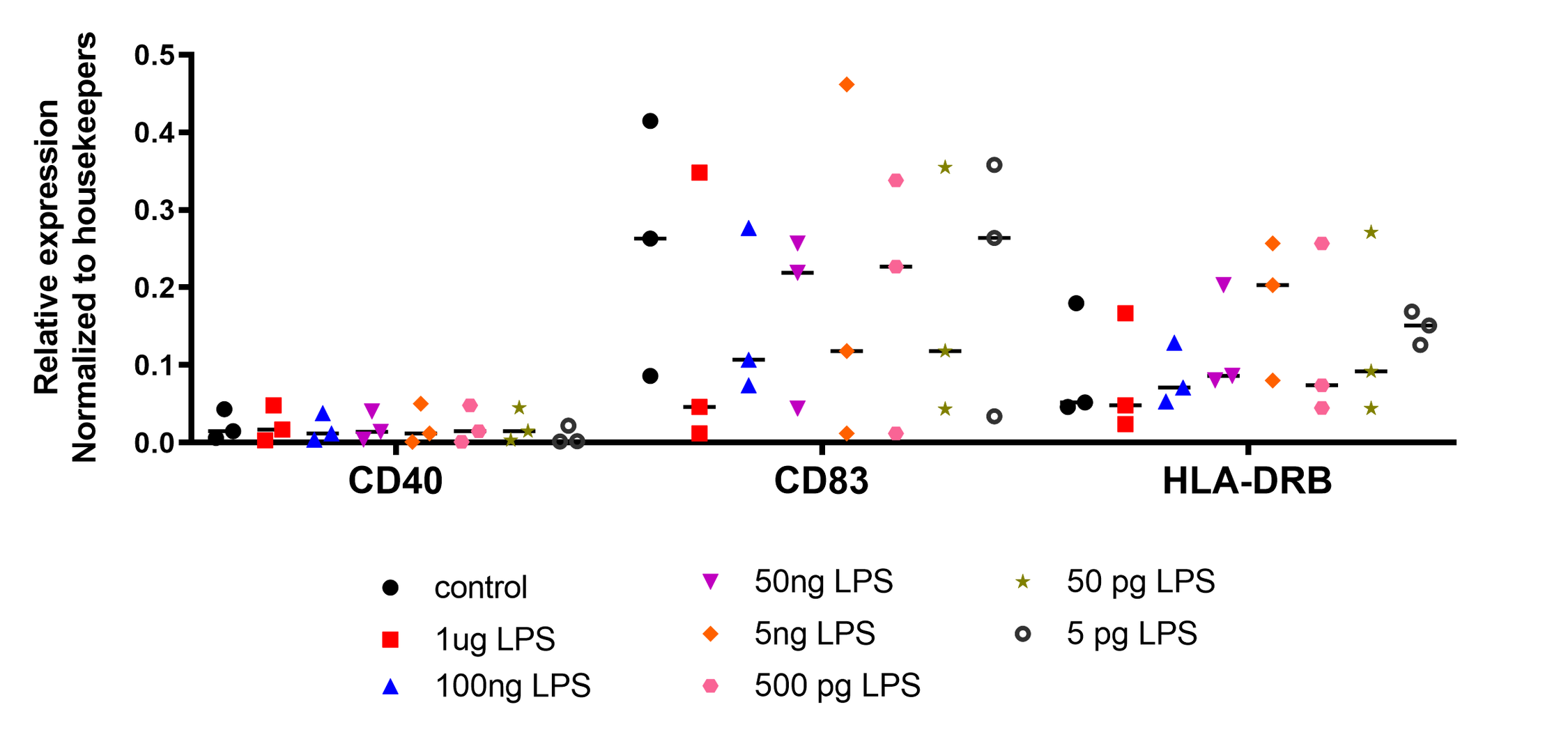

Supplement: S2 Fig — Quantitative RT-PCR failed to reveal any increased transcription of CD40, CD83, or HLA-DR after 24 hours of exposure to varying concentrations of LPS. mRNA expression levels were normalized to housekeeper genes EIF2E2 and TBP. (TIF) [file pone.0240319.s002.tif]
