# Supplementary material for: Proinflammatory cytokines and ARDS pulmonary edema fluid induce CD40 on human mesenchymal stromal cells—A potential mechanism for immune modulation
Source: PLoS One. 2020 Oct 6;15(10):e0240319. doi: 10.1371/journal.pone.0240319 (PMC7537876; doi:10.1371/journal.pone.0240319)
Supplement: S1 Appendix — (DOCX) [file pone.0240319.s003.docx]

**S1 Appendix**

**Data underlying figures**

Data underlying Figure 1 (individual points with paired descriptive statistics). For figure A, data for CD80 and CD86 are shown in the paired tables; the remaining data is duplicated in panels B-F**.**

A

|  | CD80 Control | CD80 CytoMix | CD86 Control | CD86 CytoMix |
| --- | --- | --- | --- | --- |
| IRM8013 | 0.0012 | 0.0019 | 0.0014 | 0.0015 |
| IRM8011 | 0.0015 | 0.0034 | 0.0027 | 0.0048 |
| IRM8004 | 0.0012 | 0.0026 | 0.0012 | 0.0015 |
| UM01 | 0.0011 | 0.0018 | 0.0019 | 0.0018 |
| IRM8001 | 0.0022 | 0.0053 | 0.0207 | 0.0396 |

|  | CD80  Control | CD80  CytoMix | CD86  Control | CD85  CytoMix |
| --- | --- | --- | --- | --- |
| Min | 0.001 | 0.002 | 0.001 | 0.006 |
| 25%tile | 0.001 | 0.002 | 0.001 | 0.002 |
| Median | 0.001 | 0.003 | 0.002 | 0.002 |
| 75%tile | 0.002 | 0.004 | 0.012 | 0.022 |
| Max | 0.002 | 0.005 | 0.021 | 0.040 |
|  |  |  |  |  |
| Mean | 0.001440 | 0.003 | 0.006 | 0.010 |
| Std dev | 0.0004506 | 0.001 | 0.008 | 0.017 |
| SEM | 0.0002015 | 0.001 | 0.004 | 0.007 |

B

| **Quantitative RT-PCR data for CD40 (normalized to housekeeper TBP and EIF2E2)** | | | | | | | | |
| --- | --- | --- | --- | --- | --- | --- | --- | --- |
|  | Control | CytoMix | TNFα | IL-1β | IFN-γ | TNFα + IL1β | TNFα + IFN γ | IFN γ + IL1β |
| BSRI01 | 0.008 | 0.55 | 0.012 | 0.008 | 0.07 | 0.024 | 0.373 | 0.197 |
| IRM8013 | 0.008 | 0.981 | 0.021 | 0.024 | 0.092 | 0.041 | 0.499 |  |
| IRM8011 | 0.006 | 1.927 | 0.005 | 0.026 | 0.075 | 0.031 | 0.546 | 0.36 |
| IRM8004 | 0.008 | 1.04 | 0.022 | 0.12 | 0.109 | 0.124 | 0.824 | 0.994 |
| UM01 | 0.002 | 0.524 | 0.003 | 0.008 | 0.041 | 0.017 | 0.335 | 0.095 |
| IRM8001 | 0.009 | 1.794 | 0.056 | 0.139 | 0.11 | 0.06 | 2.18 | 0.056 |

| **CD40 summary statistics** | | | | | | | | |
| --- | --- | --- | --- | --- | --- | --- | --- | --- |
|  | Control | CytoMix | TNFα | IL-1β | IFN-γ | TNFα + IL1β | TNFα + IFN γ | IFN γ + IL1β |
| Min | 0.002 | 0.524 | 0.003 | 0.008 | 0.041 | 0.017 | 0.335 | 0.056 |
| 25%tile | 0.005 | 0.544 | 0.005 | 0.008 | 0.063 | 0.022 | 0.364 | 0.076 |
| Median | 0.008 | 1.011 | 0.017 | 0.025 | 0.084 | 0.036 | 0.522 | 0.197 |
| 75%tile | 0.008 | 1.827 | 0.031 | 0.125 | 0.109 | 0.076 | 1.163 | 0.677 |
| Max | 0.009 | 1.927 | 0.056 | 0.139 | 0.110 | 0.124 | 2.180 | 0.994 |
|  |  |  |  |  |  |  |  |  |
| Mean | 0.007 | 1.136 | 0.020 | 0.054 | 0.083 | 0.050 | 0.793 | 0.340 |
| Std dev | 0.003 | 0.602 | 0.019 | 0.059 | 0.026 | 0.039 | 0.701 | 0.384 |
| SEM | 0.001 | 0.246 | 0.008 | 0.024 | 0.011 | 0.016 | 0.286 | 0.172 |

C

| **Quantitative RT-PCR data for CD83 (normalized to housekeeper TBP and EIF2E2)** | | | | | | | | |
| --- | --- | --- | --- | --- | --- | --- | --- | --- |
|  | Control | CytoMix | TNFα | IL-1β | IFN-γ | TNFα + IL1β | TNFα + IFN γ | IFN γ + IL1β |
| BSRI01 | 0.113 | 5.4 | 2.207 | 0.14 | 0.184 | 0.109 | 5.714 | 0.728 |
| IRM8013 | 0.084 | 2.221 | 0.35 | 0.088 | 0.09 | 0.08 | 4.939 | 0.182 |
| IRM8011 | 0.348 | 15.539 | 0.261 | 0.223 | 0.176 | 0.229 | 4.17 | 1.364 |
| IRM8004 | 0.07 | 8.731 | 0.336 | 0.239 | 0.064 | 0.391 | 4.735 | 1.008 |
| UM01 | 0.032 | 4.029 | 0.098 | 0.03 | 0.043 | 0.05 | 2.683 | 0.098 |
| IRM8001 | 0.133 | 29.066 | 0.895 | 0.562 | 0.12 | 0.89 | 17.76 | 0.895 |

| **CD83 Summary Statistics** | | | | | | | | |
| --- | --- | --- | --- | --- | --- | --- | --- | --- |
|  | Control | CytoMix | TNFα | IL-1β | IFN-γ | TNFα + IL1β | TNFα + IFN γ | IFN γ + IL1β |
| Min | 0.032 | 2.221 | 0.098 | 0.030 | 0.043 | 0.050 | 2.683 | 0.098 |
| 25%tile | 0.061 | 3.577 | 0.220 | 0.074 | 0.059 | 0.073 | 3.798 | 0.161 |
| Median | 0.099 | 7.066 | 0.343 | 0.182 | 0.105 | 0.169 | 4.837 | 0.812 |
| 75%tile | 0.187 | 18.92 | 1.223 | 0.320 | 0.178 | 0.516 | 8.726 | 1.097 |
| Max | 0.348 | 29.07 | 2.207 | 0.562 | 0.184 | 0.890 | 17.76 | 1.364 |
|  |  |  |  |  |  |  |  |  |
| Mean | 0.130 | 10.83 | 0.691 | 0.214 | 0.113 | 0.292 | 6.667 | 0.713 |
| Std dev | 0.112 | 10.09 | 0.790 | 0.182 | 0.058 | 0.319 | 5.528 | 0.491 |
| SEM | 0.046 | 4.119 | 0.322 | 0.077 | 0.024 | 0.130 | 2.257 | 0.200 |

D

| **Quantitative RT-PCR data for HLA-DRB (normalized to housekeeper TBP and EIF2E2)** | | | | | | | | |
| --- | --- | --- | --- | --- | --- | --- | --- | --- |
|  | Control | CytoMix | TNFα | IL-1β | IFN-γ | TNFα + IL1β | TNFα + IFN γ | IFN γ + IL1β |
| BSRI01 | 0.002 | 2.69 | 0.043 | 0.037 | 8.012 | 0.056 | 5.06 | 6.324 |
| IRM8013 | 0.024 | 3.974 | 0.096 | 0.14 | 5.909 | 0.002 | 1.788 | 7.078 |
| IRM8011 | 0.524 | 2.794 | 0.077 | 0.07 | 1.4 | 0.237 | 1.966 | 4.053 |
| IRM8004 | 0.278 | 0.047 | 0.033 | 0.053 | 0.037 | 0.074 | 0.054 | 0.055 |
| UM01 | 0.016 | 4.022 | 0.042 | 0.043 | 5.296 | 0.134 | 7.02 | 3.725 |
| IRM8001 | 0.042 | 4.65 | 0.068 | 0.035 | 1.54 | 0.07 | 4.11 | 0.068 |

| **HLA-DRB summary statistics** | | | | | | | | |
| --- | --- | --- | --- | --- | --- | --- | --- | --- |
|  | Control | CytoMix | TNFα | IL-1β | IFN-γ | TNFα + IL1β | TNFα + IFN γ | IFN γ + IL1β |
| Min | 0.002 | 0.047 | 0.033 | 0.035 | 0.037 | 0.002 | 0.054 | 0.055 |
| 25%tile | 0.013 | 2.029 | 0.040 | 0.037 | 1.059 | 0.043 | 1.355 | 0.065 |
| Median | 0.033 | 3.384 | 0.056 | 0.048 | 3.418 | 0.072 | 3.038 | 3.889 |
| 75%tile | 0.340 | 4.179 | 0.082 | 0.088 | 6.435 | 0.160 | 5.550 | 6.513 |
| Max | 0.524 | 4.650 | 0.096 | 0.140 | 8.012 | 0.237 | 7.020 | 7.078 |
|  |  |  |  |  |  |  |  |  |
| Mean | 0.148 | 3.030 | 0.060 | 0.063 | 3.699 | 0.096 | 3.333 | 3.551 |
| Std dev | 0.212 | 1.647 | 0.024 | 0.040 | 3.143 | 0.081 | 2.537 | 2.992 |
| SEM | 0.086 | 0.673 | 0.010 | 0.016 | 1.283 | 0.033 | 1.036 | 1.222 |

E

| **Quantitative RT-PCR data for TSG6 (normalized to housekeeper TBP and EIF2E2)** | | | | | | | | |
| --- | --- | --- | --- | --- | --- | --- | --- | --- |
|  | Control | CytoMix | TNFα | IL-1β | IFN-γ | TNFα + IL1β | TNFα + IFN γ | IFN γ + IL1β |
| BSRI01 | 0.366 | 377.93 | 6.872 | 45.783 | 1.708 | 56.592 | 308.259 | 145.01 |
| IRM8013 | 0.98 | 416.433 | 11.071 | 25.399 | 1.513 | 165.742 | 5.418 | 105.724 |
| IRM8011 | 20.909 | 291.668 | 42.054 | 33.52 | 32.583 | 42.954 | 73.884 | 52.186 |
| IRM8004 | 0.288 | 140.486 | 1.288 | 2.208 | 0.5 | 8.484 | 34.322 | 20.532 |
| UM01 | 0.588 | 148.729 | 0.792 | 1.138 | 0.355 | 8.699 | 51.872 | 6.737 |
| IRM8001 | 1.53 | 237.465 | 13.964 | 18.092 | 3.58 | 13.96 | 140.34 | 13.964 |

| **TSG6 Summary Statistics** | | | | | | | | |
| --- | --- | --- | --- | --- | --- | --- | --- | --- |
|  | Control | CytoMix | TNFα | IL-1β | IFN-γ | TNFα + IL1β | TNFα + IFN γ | IFN γ + IL1β |
| Min | 0.288 | 140.5 | 0.792 | 1.138 | 0.3550 | 8.484 | 5.418 | 6.737 |
| 25%tile | 0.347 | 146.7 | 1.164 | 1.941 | 0.4638 | 8.645 | 27.10 | 12.16 |
| Median | 0.784 | 264.6 | 8.972 | 21.75 | 1.611 | 28.46 | 62.88 | 36.36 |
| 75%tile | 6.375 | 387.6 | 20.99 | 36.59 | 10.83 | 83.88 | 182.3 | 115.5 |
| Max | 20.91 | 416.4 | 42.05 | 45.78 | 32.58 | 165.7 | 308.3 | 145.0 |
|  |  |  |  |  |  |  |  |  |
| Mean | 4.110 | 268.8 | 12.67 | 21.02 | 6.707 | 49.41 | 102.3 | 57.36 |
| Std dev | 8.242 | 115.0 | 15.31 | 17.59 | 12.73 | 60.36 | 110.7 | 56.30 |
| SEM | 3.365 | 46.94 | 6.250 | 7.180 | 5.197 | 24.64 | 45.18 | 22.99 |

F

| **Quantitative RT-PCR data for PTGS2 (normalized to housekeeper TBP and EIF2E2)** | | | | | | | | |
| --- | --- | --- | --- | --- | --- | --- | --- | --- |
|  | Control | CytoMix | TNFα | IL-1β | IFN-γ | TNFα + IL1β | TNFα + IFN γ | IFN γ + IL1β |
| BSRI01 | 0.115 | 80.36 | 4.032 | 16.081 | 0.796 | 63.304 | 23.542 | 28.922 |
| IRM8013 | 0.118 | 68.117 | 0.717 | 2.919 | 0.36 |  | 11.13 |  |
| IRM8011 | 0.366 | 377.93 | 6.872 | 45.783 | 1.708 | 56.592 | 308.259 | 145.01 |
| IRM8004 | 0.304 | 32.461 | 1.41 | 8.114 | 1.216 | 30.606 | 15.941 | 17.35 |
| UM01 | 0.13 | 31.452 | 0.099 | 1.051 | 0.173 | 4.244 | 1.544 | 3.013 |
| IRM8001 | 0.506 | 66.481 | 0.869 | 17.828 | 3.4 | 0.87 | 4.38 | 0.869 |

| **PTGS2 summary statistics** | | | | | | | | |
| --- | --- | --- | --- | --- | --- | --- | --- | --- |
|  | Control | CytoMix | TNFα | IL-1β | IFN-γ | TNFα + IL1β | TNFα + IFN γ | IFN γ + IL1β |
| Min | 0.115 | 31.45 | 0.099 | 1.051 | 0.173 | 0.870 | 1.544 | 0.869 |
| 25%tile | 0.117 | 32.21 | 0.563 | 2.452 | 0.313 | 2.557 | 3.671 | 1.941 |
| Median | 0.217 | 67.30 | 1.140 | 12.10 | 1.006 | 30.61 | 13.54 | 17.35 |
| 75%tile | 0.401 | 154.8 | 4.742 | 24.82 | 2.131 | 59.95 | 94.72 | 86.97 |
| Max | 0.506 | 377.9 | 6.872 | 45.78 | 3.400 | 63.30 | 308.3 | 145.0 |
|  |  |  |  |  |  |  |  |  |
| Mean | 0.257 | 109.5 | 2.333 | 15.30 | 1.276 | 31.12 | 60.80 | 39.03 |
| Std dev | 0.162 | 133.0 | 2.611 | 16.40 | 1.182 | 28.82 | 121.5 | 60.33 |
| SEM | 0.066 | 54.31 | 1.066 | 6.694 | 0.483 | 12.89 | 49.60 | 26.98 |

**Table of p values corresponding to Figure 1**

|  | Kruskal-Wallis ANOVA | Mann-Whitney p values (compared to control) | | | | | | |
| --- | --- | --- | --- | --- | --- | --- | --- | --- |
|  |  | CytoMix | TNFα | IL-1β | IFN-γ | TNFα + IL1β | TNFα + IFN γ | IFN γ + IL1β |
| CD40 | <0.001 | 0.002 | 0.236 | 0.035 | 0.002 | 0.002 | 0.002 | 0.004 |
| CD83 | <0.001 | 0.002 | 0.041 | 0.394 | 0.937 | 0.485 | 0.002 | 0.026 |
| HLA-DR | 0.003 | 0.009 | 0.509 | 0.589 | 0.015 | 0.738 | 0.009 | 0.026 |
| TSG6 | <0.001 | 0.002 | 0.132 | 0.026 | 0.485 | 0.015 | 0.004 | 0.015 |
| PTGS2 | <0.001 | 0.002 | 0.065 | 0.002 | 0.041 | 0.004 | 0.002 | 0.004 |

| **Detailed quantification of Figure 2** | | | | |
| --- | --- | --- | --- | --- |
| Cell line | % CD40 positive  Control | % CD40 positive  CytoMix | CD40 MFI  Control | CD40 MFI  CytoMix |
| IRM8004 | 1.85 | 45.8 | 1.51 ± 8.91 | 13.1 ± 62.6 |
| IRM8013 | 5.67 | 60.6 | 2.32 ± 14.8 | 26.5 ± 118 |

| **Detailed quantification of Figure 3** | | | | |
| --- | --- | --- | --- | --- |
| Panel | Description | Control  # Cells Positive/#Cells counted  (% positive) | Cytomix  # Cells Positive/#Cells counted  (% positive) | Fisher’s exact  p value |
| A | FABP4 staining (adipocytes) | 51/144  (35.4%) | 7/127  (5.5%) | < 0.001 |
| B | Oil Red O staining  (adiopocytes) | 73/103  (70.9%) | 18/108  (16.7%) | < 0.001 |
| C | Aggrecan staining  (chondrocytes) | 8/113  (7.1%) | 9/113  (8.0%) | 1.000 |
| D | Osteocalcin staining  (osteocytes) | 114/131  (87.0%) | 83/99  (83.8%) | 0.570 |

**Pulmonary Edema Fluid Experimental Data**

| **Quantitative RT-PCR data associated with pulmonary edema fluid studies (normalized to housekeeper TBP and EIF2E2)** | | | | |
| --- | --- | --- | --- | --- |
|  | **Control** | **CytoMix** | **Hydrostatic pulmonary edema fluid** | **ARDS pulmonary edema fluid** |
| **CD40** |  |  |  |  |
| IRM8013 | 0.007 | 2.61 | 0.031 | 0.033 |
| IRM8004 | 0.003 | 0.916 | 0.004 | 0.006 |
| BSRI01 | 0.005 | 0.961 | 0.013 | 0.02 |
| UM01 | 0.006 | 1.783 | 0.019 | 0.04 |
| **CD83** |  |  |  |  |
| IRM8013 | 0.127 | 20.492 | 0.251 | 0.124 |
| IRM8004 | 0.089 | 14.835 | 0.07 | 0.107 |
| BSRI01 | 0.138 | 7.457 | 0.213 | 0.091 |
| UM01 | 0.057 | 6.912 | 0.086 | 0.086 |
| **HLA-DRB** |  |  |  |  |
| IRM8013 | 0.043 | 0.666 | 0.094 | 0.055 |
| IRM8004 | 0.033 | 3.755 | 0.027 | 0.058 |
| BSRI01 | 0.088 | 0.998 | 0.044 | 0.181 |
| UM01 | 0.227 | 0.224 | 0.301 | 0.351 |
| **TSG6** |  |  |  |  |
| IRM8013 | 0.488 | 663.493 | 0.969 | 3.899 |
| IRM8004 | 0.388 | 499.012 | 0.256 | 2.524 |
| BSRI01 | 0.702 | 494.571 | 0.894 | 2.775 |
| UM01 | 0.11 | 112.437 | 0.175 | 0.689 |
| **PTGS2** |  |  |  |  |
| IRM8013 | 0.138 | 83.805 | 3.935 | 15.601 |
| IRM8004 | 0.336 | 132.197 | 4.235 | 44.186 |
| BSRI01 | 0.348 | 65.098 | 6.618 | 11.225 |
| UM01 | 0.127 | 6.993 | 5.618 | 21.968 |

| **Table of p values for pulmonary edema fluid experiments** | | | | |
| --- | --- | --- | --- | --- |
|  | Kruskal-Wallis  p value | Post hoc unpaired *t* test (two tailed) compared to control | | |
|  |  | CytoMix | Hydrostatic pulmonary edema fluid | ARDS  pulmonary edema fluid |
| CD40 | 0.013 | 0.008 | 0.091 | 0.042 |
| CD83 | 0.019 | 0.009 | 0.326 | 0.972 |
| HLA-DRB | 0.055 | --- | --- | --- |
| TSG6 | < 0.001 | 0.009 | 0.554 | 0.023 |
| PTGS2 | < 0.001 | 0.032 | < 0.001 | 0.020 |

**Data underlying S1 Fig**

**
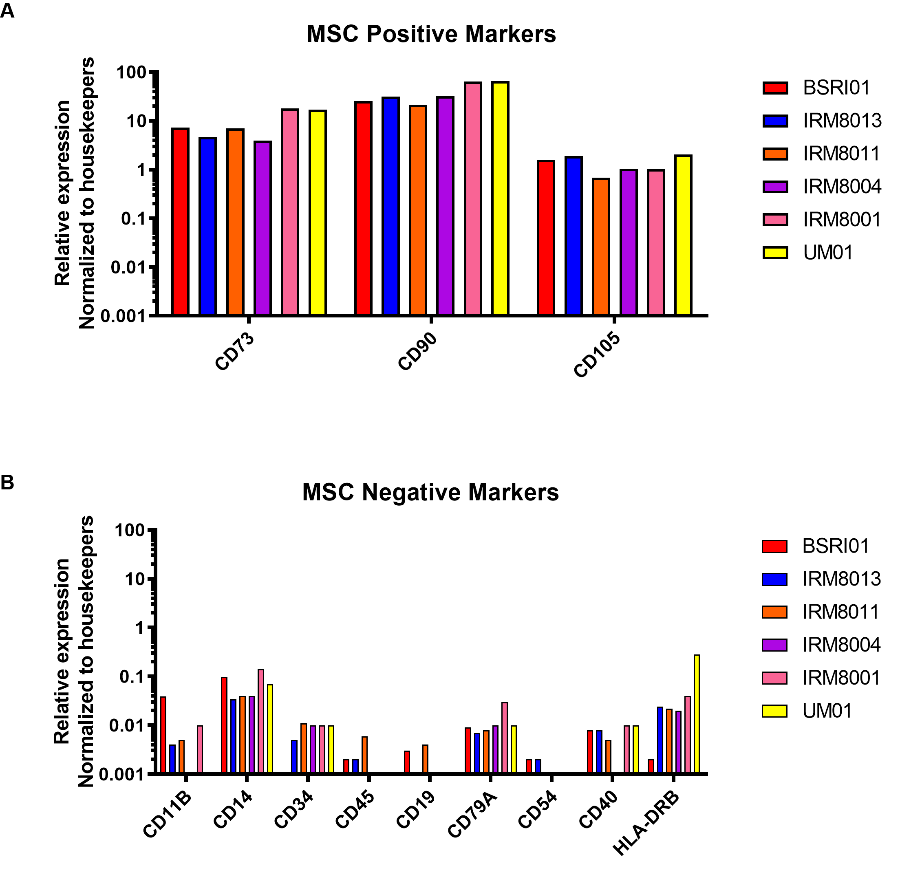
**

| **Quantitative RT-PCR data for validation of hMSC phenotype (normalized to housekeeper TBP and EIF2E2)** | | | | | | |
| --- | --- | --- | --- | --- | --- | --- |
|  | BSRI01 | IRM8013 | IRM8011 | IRM8004 | IRM8001 | UM01 |
| CD73 | 7.307 | 4.695 | 6.945 | 3.93 | 17.88 | 16.85 |
| CD90 | 25.417 | 31.27 | 21.235 | 32.15 | 64.16 | 65.4 |
| CD105 | 1.592 | 1.877 | 0.676 | 1.04 | 1.02 | 2.04 |
| CD11B | 0.039 | 0.004 | 0.005 | 0 | 0.01 | 0 |
| CD14 | 0.098 | 0.034 | 0.04 | 0.04 | 0.14 | 0.07 |
| CD34 | 0.001 | 0.005 | 0.011 | 0.01 | 0.01 | 0.01 |
| CD45 | 0.002 | 0.002 | 0.006 | 0 | 0 | 0 |
| CD19 | 0.003 | 0.001 | 0.004 | 0 | 0 | 0 |
| CD79A | 0.009 | 0.007 | 0.008 | 0.01 | 0.03 | 0.01 |
| CD54 | 0.002 | 0.002 | 0.001 | 0 | 0 | 0 |
| CD40 | 0.008 | 0.008 | 0.005 | 0 | 0.01 | 0.01 |

**Data underlying S2 Fig**

**
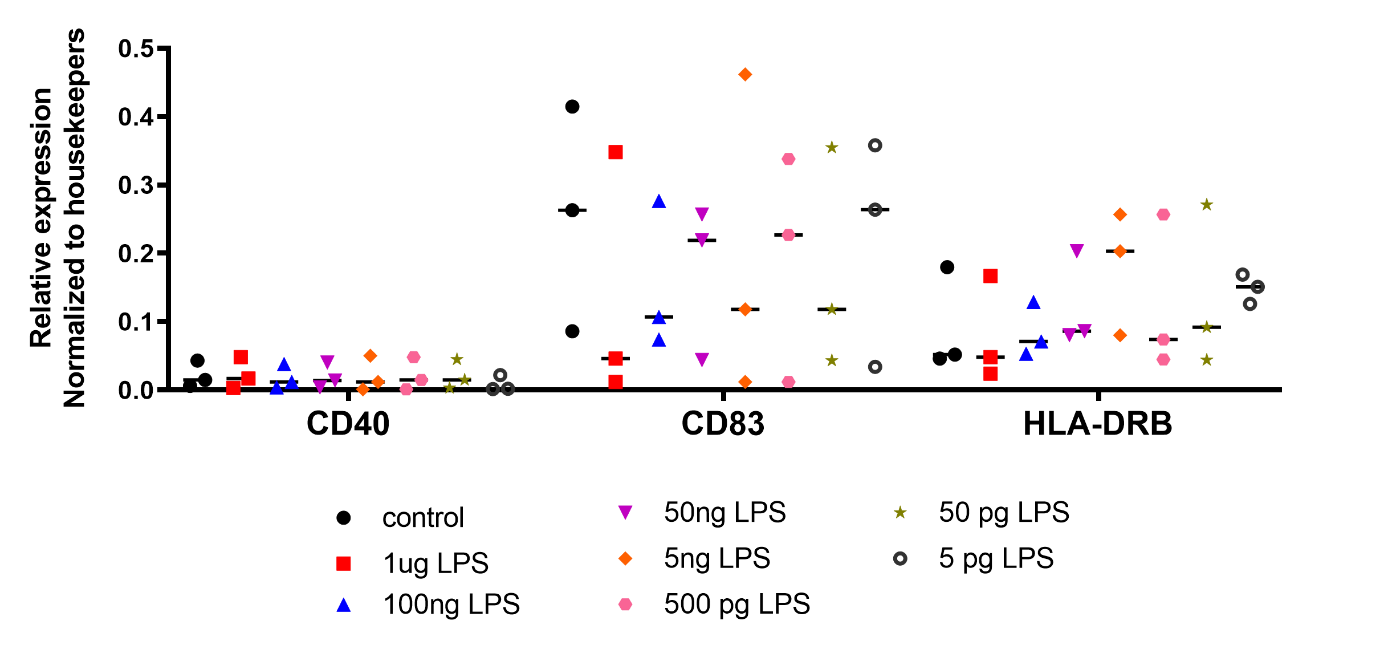
**

|  |  | | |  | | |  | | |  | | |  | | |  | | | One way ANOVA |
| --- | --- | --- | --- | --- | --- | --- | --- | --- | --- | --- | --- | --- | --- | --- | --- | --- | --- | --- | --- |
| CD40 | 0.015 | 0.043 | 0.006 | 0.017 | 0.048 | 0.003 | 0.012 | 0.038 | 0.004 | 0.014 | 0.04 | 0.004 | 0.012 | 0.05 | 0.001 | 0.015 | 0.048 | 0.001 | 0.993 |
| CD83 | 0.263 | 0.415 | 0.086 | 0.012 | 0.348 | 0.046 | 0.107 | 0.277 | 0.074 | 0.219 | 0.257 | 0.044 | 0.118 | 0.462 | 0.012 | 0.227 | 0.338 | 0.012 | 0.991 |
| HLA-DRB | 0.046 | 0.052 | 0.18 | 0.024 | 0.048 | 0.167 | 0.053 | 0.071 | 0.129 | 0.086 | 0.08 | 0.203 | 0.257 | 0.08 | 0.203 | 0.257 | 0.074 | 0.045 | 0.528 |
