# Supplementary material for: Proinflammatory cytokines and ARDS pulmonary edema fluid induce CD40 on human mesenchymal stromal cells—A potential mechanism for immune modulation
Source: PLoS One. 2020 Oct 6;15(10):e0240319. doi: 10.1371/journal.pone.0240319 (PMC7537876; doi:10.1371/journal.pone.0240319)
Supplement: Attachment — Submitted filename: Response to Reviewers.docx [file pone.0240319.s004.docx]

September 18, 2020

To: Editors, PLOS ONE

Attached, please find our revised original research manuscript, entitled “Proinflammatory cytokines and ARDS pulmonary edema fluid induce CD40 on human mesenchymal stromal cells – a mechanism for immune modulation.” We are most grateful to the reviewer’s for their thoughtful comments and have responded these below. We appreciate the Editors allowing us to submit this revised manuscript.

**Editorial Office:**

1. Please ensure that your manuscript meets PLOS ONE's style requirements, including those for file naming. The PLOS ONE style templates can be found at https://journals.plos.org/plosone/s/file?id=wjVg/PLOSOne_formatting_sample_main_body.pdf and https://journals.plos.org/plosone/s/file?id=ba62/PLOSOne_formatting_sample_title_authors_affiliations.pdf

This has been done.

2. We note that you have included the phrase “data not shown” in your manuscript. Unfortunately, this does not meet our data sharing requirements. PLOS does not permit references to inaccessible data. We require that authors provide all relevant data within the paper, Supporting Information files, or in an acceptable, public repository. Please add a citation to support this phrase or upload the data that corresponds with these findings to a stable repository (such as Figshare or Dryad) and provide and URLs, DOIs, or accession numbers that may be used to access these data. Or, if the data are not a core part of the research being presented in your study, we ask that you remove the phrase that refers to these data.

We apologize for this oversight. This data has been added as a supplemental figure. We have also expanded the statistical analysis section and added a new S3 data appendix to improve data transparency in accordance with your statistical reporting guidelines.

**Reviewer #1:**

This observational paper describes some interesting phenotypes in hMSCs under inflammatory conditions, including the upregulation of CD40 by inflammatory cytokines or ARDS edema fluid. Given the potential for hMSC treatment in ARDS patients, these important findings could lead to further investigations of the regulation of hMSC function and efficacy in ARDS. However, given that the current data does not comprehensively describe any novel mechanistic regulation of hMSC function by each of the different inflammatory stimuli, the authors could include some further studies/explanations to further enhance the insight provided by their work.

MAJOR
1. Which cell markers were used to define the hMSCs? Given that the cells were obtained from 3 different locations/sources, how did the authors ensure that the cells were consistent?

We felt that using hMSCs from multiple sources demonstrated that the phenotype described was consistent and robust for , increasingly the likelihood of reproducibility if studies were to be repeated in other laboratories. Additionally, this avoided the problem of a finding that might be reflective of cells from a specific location/source, rather than broadly applicable to hMSCs. All cell lines were validated using qPCR for the presence of CD73, CD90, and CD105, as well as the absence of CD11b, CD14, CD34, CD45, CD19, CD79A, CD54, and HLADRB. This data is shown in supplemental figure S1. This has been added to the methods. (Lines 80-82)

2. Regarding the different cytokine combinations, text in the introduction states that secretion of IL1b, TNFa, IL6, and IL8 is increased. Later in the introduction, the cytomix of TNFa, IFNy, and IL1b is mentioned. In the methods, the cytomix is described and another stimulus is introduced, i.e. LPS. Do the concentrations of cytokines and LPS used compare similarly to the circulating levels found in ARDS patient samples? Data describing these levels would be useful. Alternatively, authors could provide a clear rationale describing why the respective cytokines and concentrations were used.

The CytoMix cytokine cocktail was selected as it has been shown to recapitulate the effects of acute lung injury pulmonary edema fluid on alveolar type II cells. This sentence has been added to the introduction. (Lines 66-69)

3. Similarly, were the cytokine levels measured in the hydrostatic and ARDS edema fluids? These levels could be provided for comparison, along with suggestions for future studies that would help elucidate the different mechanisms responsible for how/why ARDS but not hydrostatic edema fluid resulted in increased CD40 expression.

We agree that measure the cytoking levels in hydrostatic and ARDS edema fluids could be information. Unfortunately, due to very limited sample availability, cytokine levels from these pulmonary edema fluid samples were not measured.

4. In terms of the potential for treatment of ARDS using hMSCs, and given the pluripotency demonstrated, which cell phenotype do the authors expect to be most beneficial? It may be useful to the reader if the authors could add data or an explanation describing how this immunomodulatory phenotype could be maintained in clinical studies, especially given that the cytokine levels in human ARDS will likely vary over time and between individuals at different stages of disease progression.

Thank you for this recommendation. We have modified the discussion to say, “Given the robust nature of this phenotype under specific conditions and the variability among human disease states, preconditioning with CytoMix to induce an immunomodulatory phenotype may be important to ensure a uniform hMSC exposure in clinical trials, but this will require additional *in vivo* investigations.” (Lines 282-284) At present, we have not conducted any *in vivo* clinical trials to look at these immunomodulatory phenotypes after administration.

5. It would be useful to quantify the findings in Figure 3.

Thank you for this suggestion. These have been quantified within the Figure 3 legend, within the text, and in the S3 data appendix.

MINOR
6. A few sentences in the Introduction would benefit from references being added. For example, “Alveolar macrophage activation leads to secretion of inflammatory cytokines, including interleukin-1β (IL-1β), tumor necrosis factor (TNF), interleukin-6 (IL-6), and interleukin-8 (IL-8), which lead to additional neutrophil activation.”, “hMSCs have many beneficial immunomodulatory effects.”, and “Through soluble mediators such as indoleamine-2,3-dioxygenase and PGE2, hMSCs promote the M1 to M2 transition of activated macrophages.”

Thank you for this suggestion. Additional references have been added. The sentence stating ‘hMSCs have many beneficial immunomodulatory effects” is an introductory sentence to summarize the remainder of that paragraph that delineates these benefits.

7. Regarding the hydrostatic pulmonary edema fluid, it may not be clear to the reader from whom this fluid was taken. For example, what were the demographics of these individuals/patients?

While this would indeed be interesting, this information is not available.

8. For consistency, the heading “Flow cytometry – “ in the Methods could be replaced with “Flow cytometry.”, and “CD40 is expressed on the cell surface after CytoMix Exposure” replaced with “CD40 is expressed on the cell surface after CytoMix exposure”.

These changes have been made.

9. For clarity, regarding the following sentence in the Discussion, it may be useful to expand this sentence by including an explanation of the type of methods/studies that would be used to investigate/induce preconditioning: “Given the robust nature of this phenotype under specific conditions and the variability among human disease states, preconditioning may be important to ensure a uniform hMSC exposure in clinical trials.”

We have added that additional *in vivo* experiments would be required. We respectfully feel that a larger discussion would take away from the main finding of this report; namely the finding of CD40 positivity.

10. Given the observational nature of the findings, it may be more suitable and reflective of the findings to remove the following phrase from the title: ” – a mechanism for immune modulation”

We have added the word “potential” before mechanism, as these are observational studies and direct causation is not established.

11. According to the Methods, Mann Whitney or T-tests were used throughout. It is my understanding, however, that ANOVA should be used for multiple group comparisons (i.e. when there are more than 2 groups being compared).

ANOVA, or Kruskal-Wallis-given our data is non-parametric, evaluate if there are differences between any of the groups in a multiple comparison analysis, but cannot evaluate which groups were different. For the second portion of the analysis, we used Mann-Whitney U tests to ascertain between group comparisons. The Kruskal-Wallis ANOVA was <0.01 for panels B-F of Figure 2. This has been added to the caption.

Reviewer #2:

This is an interesting paper by Wilfong et al that shows upregulated CD40 on human mesenchymal stem/stromal cells (hMSCs) after exposure to proinflammatory cytokines or ARDS pulmonary edema fluid. Furthermore, the expression of several anti-inflammatory cytokines and paracrine effectors, on hMSCs was also up-regulated. Finally, proinflammatory cytokines have been shown to reduce pleuripotency of hMSCs and block hMSCs differentiation into adipocytes. Overall, this is a very well written and a complete study on the important role of hMSCs in immunomodulation in ARDS. I only have a few minor comments that will help to clarify a few points in the manuscript.

Minor comments:
1. In Figure 2 and 3, quantification is required.

We have now performed quantification for both Figure 2 and Figure 3.

2. Please check and define all abbreviations on the first occurrence in context.

We have reviewed abbreviations, and made updates as necessary to define upon first occurrence in the text.

3. In the Statistical Analysis section, The description “P < 0.05 was considered significant” is missing.

Thank you for noting this. We have expanded the statistical analysis section and included this.

Sincerely,

Erin M. Wilfong, MD PhD

Fellow, Divisions of Rheumatology, Pulmonary and Critical Care Medicine

Vanderbilt University Medical Center

Michael A. Matthay, MD

Professor of Medicine and Anesthesia

University of California, San Francisco
